# Experimental evaluation of the impact of sEMG interfaces in enhancing embodiment of virtual myoelectric prostheses

**DOI:** 10.1186/s12984-024-01352-7

**Published:** 2024-04-16

**Authors:** Theophil Spiegeler Castañeda, Mathilde Connan, Patricia Capsi-Morales, Philipp Beckerle, Claudio Castellini, Cristina Piazza

**Affiliations:** 1 Department of Computer Engineering, Technical University of Munich (TUM), Garching bei Munich, Germany; 2 Institute of Robotics and Mechatronics, German Aerospace Center (DLR), Oberpfaffenhofen, Germany; 3 Department Artificial Intelligence in Biomedical Engineering, Friedrich-Alexander-Universität Erlangen-Nürnberg (FAU), Erlangen, Germany; 4 Department of Electrical Engineering, Friedrich-Alexander-Universität Erlangen-Nürnberg (FAU), Erlangen, Germany; 5 Munich Institute of Robotics and Machine Intelligence, Technical University of Munich (TUM), Munich, Germany

**Keywords:** Upper-limb prosthesis, Embodiment, Surface EMG, Virtual reality

## Abstract

**Introduction:**

Despite recent technological advances that have led to sophisticated bionic prostheses, attaining embodied solutions still remains a challenge. Recently, the investigation of prosthetic embodiment has become a topic of interest in the research community, which deals with enhancing the perception of artificial limbs as part of users’ own body. Surface electromyography (sEMG) interfaces have emerged as a promising technology for enhancing upper-limb prosthetic control. However, little is known about the impact of these sEMG interfaces on users’ experience regarding embodiment and their interaction with different functional levels.

**Methods:**

To investigate this aspect, a comparison is conducted among sEMG configurations with different number of sensors (4 and 16 channels) and different time delay. We used a regression algorithm to simultaneously control hand closing/opening and forearm pronation/supination in an immersive virtual reality environment. The experimental evaluation includes 24 able-bodied subjects and one prosthesis user. We assess functionality with the Target Achievement Control test, and the sense of embodiment with a metric for the users perception of self-location, together with a standard survey.

**Results:**

Among the four tested conditions, results proved a higher subjective embodiment when participants used sEMG interfaces employing an increased number of sensors. Regarding functionality, significant improvement over time is observed in the same conditions, independently of the time delay implemented.

**Conclusions:**

Our work indicates that a sufficient number of sEMG sensors improves both, functional and subjective embodiment outcomes. This prompts discussion regarding the potential relationship between these two aspects present in bionic integration. Similar embodiment outcomes are observed in the prosthesis user, showing also differences due to the time delay, and demonstrating the influence of sEMG interfaces on the sense of agency.

## Introduction

Missing an upper limb not only hinders daily activities but also affects individuals’ autonomy and their active engagement in societal participation [1]. Artificial limbs aim to replace a body extremity through technological solutions, effectively mitigating significant impairments in sensory-motor capabilities and facilitating physical interaction with the environment. Recent technological progress has enabled the creation of increasingly effective artificial upper-limbs [2], which can improve the quality of life of individuals with limb loss. Despite the advanced dexterity and grasping capabilities that can be achieved by modern hand prostheses, they are often not perceived by the user as part of their own body, as described in [3, 4] as Sense of Embodiment (SoE). As the clinical translation of neuroprostheses capable of restoring lost extremities progresses towards accessibility [5], the recognition of prosthetic embodiment as a crucial factor for evaluating psycho-social outcomes becomes increasingly evident [6, 7]. Theoretically, robotic limbs can achieve high precision and strength. Nevertheless, their actual performance is substantially limited by challenges associated with bionic interfacing and bidirectional transfer of motor and sensory information between the prosthesis and the user [8].

Surface electromyography (sEMG) is the traditional interface for motor decoding and bionic interfacing, and so far the gold standard in the academic environment as well as in the clinics. The clinical standard relies on two sEMG channels and a direct control approach, with certain limitations in systems with multiple degrees of freedom (DoFs). A promising alternative is based on pattern recognition methods, that characterize the signals of muscle groups with selected features. These methods often employ 6–12 bipolar sEMG electrodes, enabling effective classification of various grasp types, which can allow more intuitive control [9, 10]. While classification techniques guarantee high robustness within each identified class, this approach hardly allows simultaneous control of multiple DoFs. To address this goal, researchers have been exploring proportional control approaches, which enable the simultaneous and proportional decoding and control of multiple DoFs through regression methods [11]. Different than classification, regression is a machine learning method that allows continuous instead of discrete predictions. Furthermore, recent development of high-density sEMG technology [12] has brought remarkable advancements regarding accuracy and decoding robustness, particularly in addressing electrode shifts. Moreover, previous research has shown that myoelectric control, i.e., provided with sEMG interfaces, and understanding of technological functions can be improved by training [13]. Among alternatives, virtual training methods have proven to be a powerful tool due to the exploration of different multi-sensory cues and immersive environments [14, 15].

Significant research has been conducted on upper-limb prostheses as essential aids for restoring both aesthetic appearance and functional autonomy. However, notable gaps persist in understanding the precise criteria required to achieve a satisfactory embodied experience [16, 17]. Embodiment has gained significant importance in prosthetics research, serving as both an indicator of technological advancements in prosthetic devices and a measure of user acceptance [6]. For instance, significant improvement in prosthesis embodiment and reduction of phantom limb pain was observed in a long-term amputee through sensory feedback delivery [18]. Although multiple definitions exist in literature, three different components for the sense of embodiment, namely *Ownership* [19], *Location* [20], and *Agency* [21], were proposed in [22] for virtual reality studies. However, capturing and quantifying this multi-dimensional concept still poses a complex challenge, especially in studies involving users in the loop (e.g [13, 23]). In the field of prostheses research, embodiment is typically approached from two perspectives: body representations and experimental phenomenology [6]. Many studies evaluate embodiment mostly relying on surveys, or in combination with other psychological measures, such as the proprioceptive drift (PPD) [24, 25]. In the context of the rubber hand illusion [26], the PPD measures the displacement between the perceived location of the users’ arm (not visible from their perspective) and the actual position. While PPD is usually conducted in physical environments, it has been also adapted into virtual setups [27]. Furthermore, other works have been exploring the sense of embodiment in immersive virtual reality through questionnaires [28–30] but without additional objective assessments (e.g. physicophysical or experimental phenomenological metrics). Note that immersive virtual reality creates a three-dimensional environment via a Head-Mounted Display and features precise tracking motion systems. Moreover, haptic interfaces may also be integrated in some occasions to offer users tactile feedback. The progress in modern control techniques opens up possibilities for improved and more adaptable bionic interfaces [31], e.g. based on machine learning algorithms, which may enhance the sense of embodiment.

This work aims to investigate the relationship between sEMG interfaces and both functionality and embodiment, including its subcomponents according with [22]. In particular, we focus on the number of sEMG sensors, time delay, and myoelectric control training. To this end, we implement a 2 degree of freedom (DoF) regressor that enables simultaneous control of hand closing/opening, and the pronation/supination of the forearm. The experimental protocol involves 24 able-bodied participants and one prosthesis user that control an upper-limb prosthesis in an immersive virtual reality environment. The controller is tested using four different conditions, including two different numbers of sEMG sensors, and a time delay to the display, similar to [32]. The sense of embodiment is quantified by an adapted version of the proprioceptive drift (PPD) test into an immersive virtual reality setup that quantifies the perception of self-location. The study includes the evaluation of subjective embodiment using a standardized survey, inspired by [32]. Additionally, functionality is assessed through the Target Achievement Control (TAC) [33]. We hypothesize that:

**H1**: *Control using a larger number of sEMG sensors yields improved functionality compared to fewer sensors.*
**H2**: *Control using a larger number of sEMG sensors enhances subjective embodiment compared to fewer sensors.*
**H3**: *Time delay reduces the embodiment of myoelectrically controlled devices.*
**H4**: *A positive relationship exists between functionality and subjective embodiment.*

## Materials and methods

**Fig. 1:**
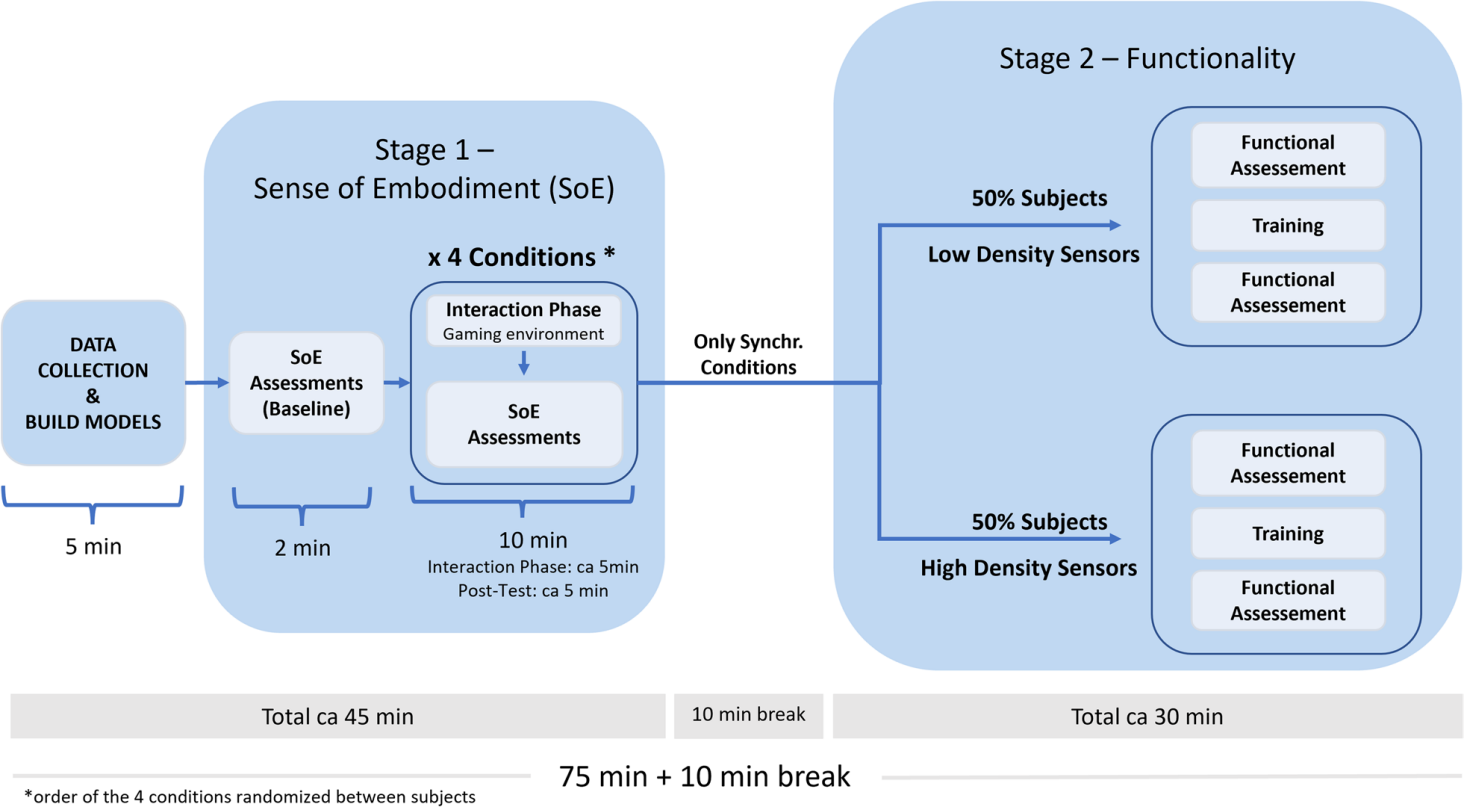
The figure shows the different sEMG configurations. The low density configuration uses channels number 1 and 5 of the first Myo armband and channels 3 and 7 from the second Myo armband. The high density condition uses all 8 channels from both Myo armbands. The alignment of the armbands on the arm can be seen on the right, for the example of a left handed person. All participants used their dominant hand during the experiments

This study evaluates the effects of different sEMG interfaces on the sense of embodiment perceived by the user and on the functionality achieved by the user. The study is designed in two stages (shown in Fig. 1), the first stage includes 2 $\times$ 2 repeated-measures for the sense of embodiment. In the second stage, which is designed as a between group study, we conduct pre- and post-training assessment for the functionality. The evaluation is conducted in an immersive virtual reality (VR) environment, where the user has a first-person visual perspective and can control a virtual prosthesis to interact with several objects. For this purpose, custom gaming and training scenes were designed to motivate and help the user familiarize with the VR environment and control strategy. The training environments are based on activities of daily living, such as moving objects with different shapes and dimensions from one table to another or opening a door. Selected assessments to measure the functionality [33] and the sense of embodiment [24, 34] have been adapted from literature and implemented in the VR environment. More details on the experimental setup, protocol, VR environments, and control method are presented in the following sections.

### Conditions

The factors included for the Sense of Embodiment (SoE) analysis are the number of sensors (*Sensors*) and the time delay (*Delay*). The interaction between them (*Sensors* $\times$ *Delay*) are our four conditions, which have been defined to observe the effect of different sEMG control interfaces on functionality and sense of embodiment. For *Sensors*, participants can experience high density (HD) or low density (LD) while for *Delay*, the virtual hand movement can be synchronous or asynchronous. Note that the terms HD and LD are contextualized within our protocol’s terminology for high and low density, respectively, and are not directly aligned with other literature uses. Specifically, the selected conditions are:

**C1**: *Asynchronous, LD*
**C2**: *Synchronous, LD*
**C3**: *Asynchronous, HD*
**C4**: *Synchronous, HD*

Where LD (low density) indicates the case where only 4 sEMG electrodes are used, and HD (high density) employs 16 electrodes. The number of 16 electrodes were chosen based on the findings of [35], which show that a number of 16 electrodes did not deteriorate the performance compared to 192 electrodes—common sensor configuration for high-density terminology, used in literature. Thus, we assumed that 16 electrodes can serve as a representative setup for high density sEMG interface. Synchronous is defined as the condition with the shortest possible delay between the input command-reading, and the execution visualized on the virtual hand. The asynchronous condition introduces a time delay of *500 ms* adopted from [32] and is supposed to be a recognizable delay [36].

### Participants

Twenty-four able-bodied participants took part in the experimental evaluation (8 female, 16 male; 3 left-hand, 21 right-hand dominant, average age of 24.1 ± 4.1 years). All participants used their dominant hand during the experiments to maximize the control performance and ensure optimal muscle condition. Nine participants had previous experience in EMG control while twelve used VR commercial games. A prosthesis user is also included in the experimental evaluation for reference and discussion regarding the applicability of the findings to the target population. This individual is a 35-year-old male with a transradial amputation of the right arm. He has no prior experience with VR or the specific myoelectric control method used in this study. All participants were naive to the specific VR environments developed and used for this study, the particular control method adopted, and the experimental protocol. All participants were in a good physical and mental condition and gave their informed consent. This study was conducted according to the guidelines of the Declaration of Helsinki, and approved by the Ethics Commission of the Technical University of Munich, reference number 478/21 S-SR (27.09.2021).

### Experimental setup

#### EMG setup and data collection

**Fig. 2:**
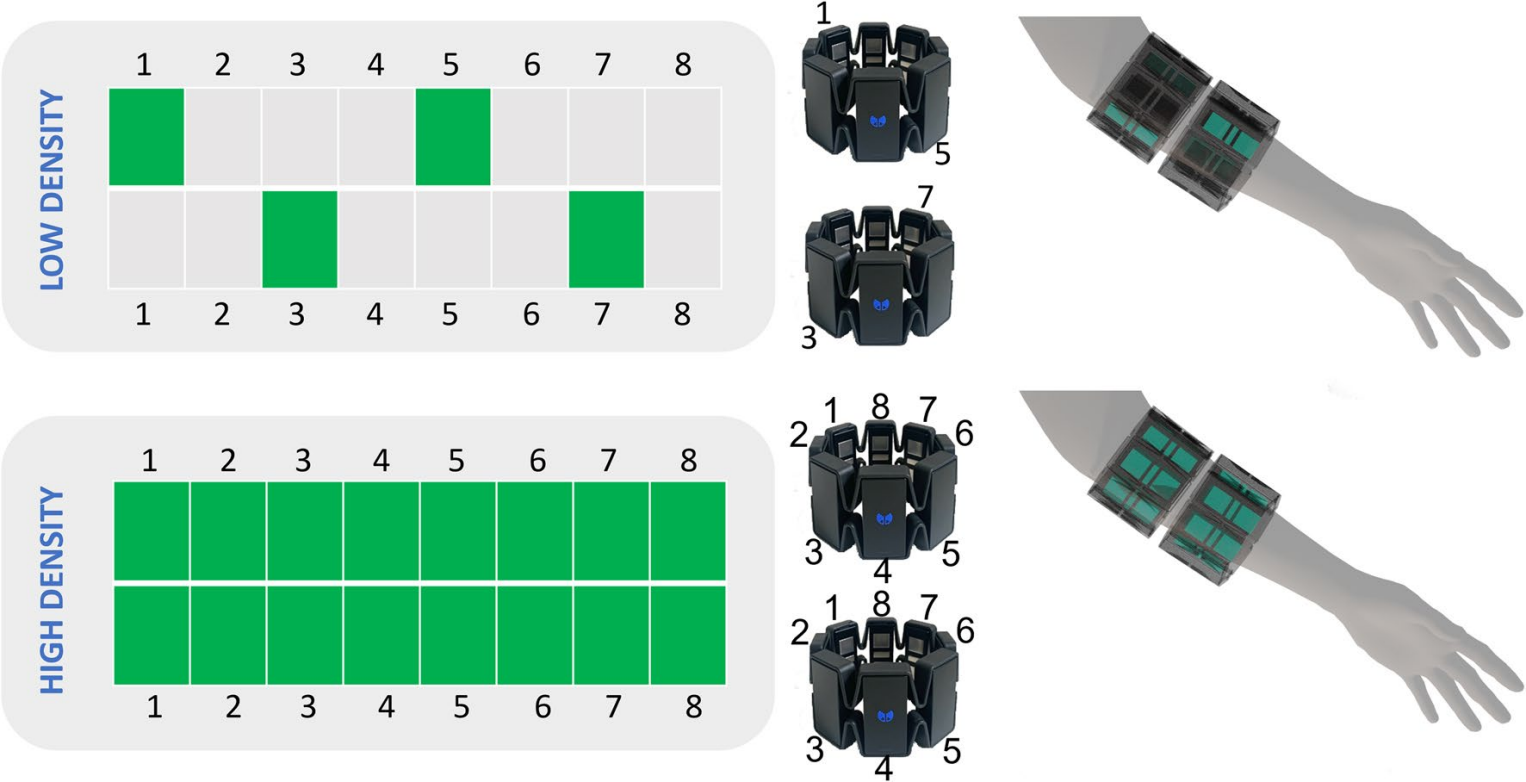
The figure shows a schematic of the experimental protocol. After giving their informed consent, the experiment starts with the sEMG data recording, and model training. The first part of the experiment focuses on the embodiment evaluation, conducting a baseline measurement, a familiarization phase in a gaming environment and a second embodiment measurment. This part of the experiment was repeated for the four conditions. After a short break, participants continue the training and functionalities assessment only with the synchronous conditions (half of them with condition **C2** while the other half with condition **C4**)

The participants were equipped with two Myo sEMG sensor armbands resulting in a total of up to 16 sensors (see Fig. 2). The armbands were placed on the muscle bulge of the participant’s forearm, approximately 1 cm below the elbow. The upper armband was placed with channel number 4 (see Fig. 2) on the extensor digitorum muscle. The sEMG sensors were located similarly for the prosthesis user, covering his entire residual limb. The sEMG data was used to control the closing and opening of the hand, as well as pro/supination of the forearm. For that purpose, the data of the sEMG armbands was acquired at 200Hz via Bluetooth Low Energy (BLE) in a C# software called *Interactive Myocontrol* developed by *DLR* for biosignal data acquisition and training. During a training phase, sEMG data were recorded while the participants were instructed to reproduce the movements of a 3D hand model, as shown on a screen in front of them (outside of immersive VR). The trained gestures were: rest, power grasp, pronation, as well as the combination of pronation and power grasp. The default position of the hand, namely the rest position, was with the palm facing upwards and hand completely opened with relaxed fingers. All trained gestures started with the default position and move to the targeted configuration. Therefore, the trained gesture of pronation covered the full range of forearm rotation, avoiding the inclusion of supination as a trained gesture. Five repetitions of this sequence of gestures were recorded: the first three in a static position, with an elbow angle of 90^∘^, and the last two dynamically, i.e., while moving the arm around to simulate VR conditions. After a second order Butterworth low-pass filter with a cut-off frequency of 1Hz, the data was then sent to a machine learning algorithm, namely Ridge Regression with Random Fourier Features (RR-RFF), which has already been used successfully online for myocontrol in several experiments [37, 38]. Once the machine learning model was trained, the prediction of the regressor was sent via User Datagram Protocol (UDP) to the VR environment in order to move a VR hand/wrist model.

#### VR setup

**Fig. 3:**
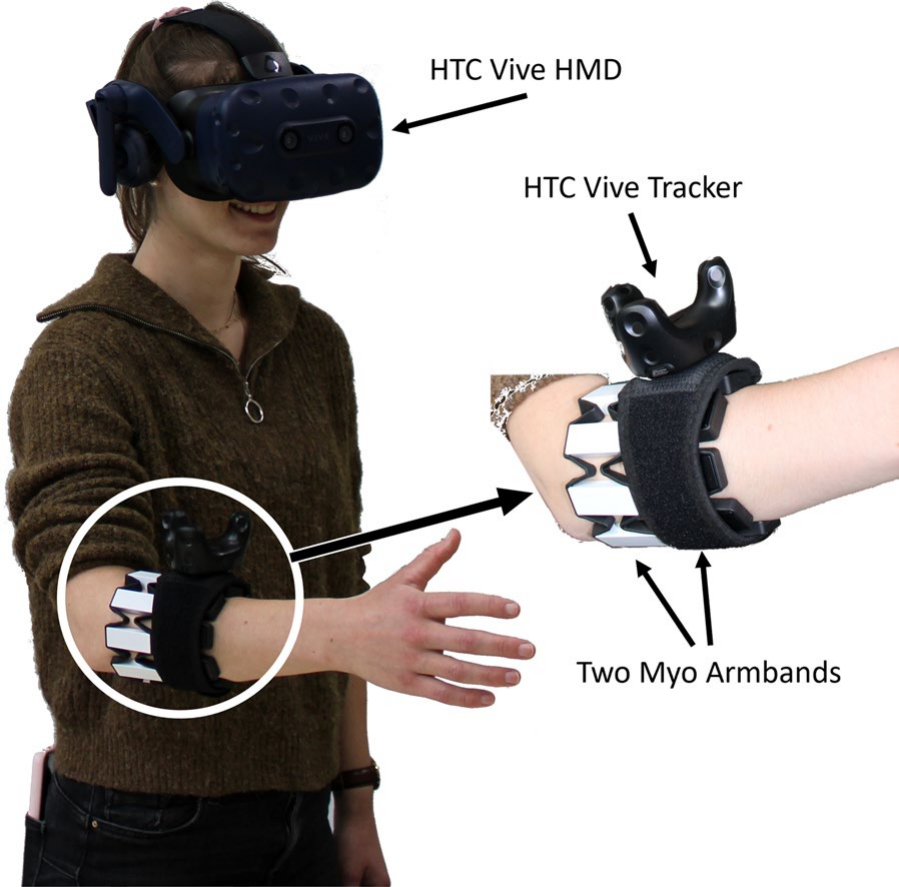
Experimental setup used in the study. The figure shows a participant wearing the *HTC Vive Pro* head mounted display (HMD), used to visualize the virtual environment. Two Myo armbands (up to 8 sEMG sensors each) and one *HTC Vive* tracker are placed on the user’s forearm

The virtual environment was created using *Unity3D* (version 2020.3.22, Unity Technologies Inc., San Francisco, USA) on a *Windows 10* operating system. The participants were using an *HTC Vive Pro* head-mounted-display (HMD) and a *HTC Vive* tracker as visualization input, and VR control. The tracker, used to detect the lower arm position, was placed on top of the more distal Myo armband, similar to [39]. The HMD was used to control the position of the shoulder, which is located 15 cm below the eye and 20 cm to the left or right (accordingly to the anthroprometric tables) of the head center. The user had a first-person visualization of one fully controllable arm, which simulates the virtual prosthesis and can be used to interact with the VR environment. The final setup of one participant wearing the HMD, the two Myo armbands (Thalmic Labs Burlington, VT, USA) and the tracker is shown in Fig. 3.

### Experimental protocol

Before starting the experiment, the participants are equipped with two sEMG Myo armbands and an *HTC Vive* tracker (HTC Corporation, Taoyuan, Taiwan) and are asked to stand in front of the computer. The experiment then begins with the sEMG data collection and model training. After, a brief visual test was conducted by the experimenter to ensure the participant is capable of performing the different movements. The participant wears the VR HMD and visualize the first VR environment. The participant is immediately guided in the first embodiment assessment environment to collect the data that will be used as baseline. The baseline represents the sense of embodiment that the user has with the least amount of time seeing and interacting with his virtual arm in VR and without any controller experience. The experiment continues with the Interaction phase, conducted in a gaming environment for 5 min, after which the sense of embodiment is evaluated. This first part of the experiment is repeated four times, one for each condition in a randomized order (the condition sequence is determined by randomized latin squares). The participant has no information about the tested condition. Once all four conditions have been evaluated, and after taking a 10-minute break, the participant continues with the second stage that focuses on the functional assessment. To limit the duration of the experiment to under 2 h and avoid fatigue, each participant experiences only one condition for the functional evaluation. Only synchronous conditions are tested since we consider them the most relevant for the functional assessment. The participants (N = 24) are divided into two equally distributed groups (N = 12), which tested condition **C2** or **C4**. Functionality is assessed before and after 10-minutes training. Five training environments were developed for the participants to experience and practice the control modality. A schematic of the whole experimental protocol is shown in Fig. 1. A prosthesis user conducted the same experimental protocol. However, time constraints and fatigue prevented him from conducting the second part of the experiment (Stage 2—Functionality). For this reason, prosthesis user results do only concern embodiment assessment.

### Virtual reality environments

The VR environment was designed to include a total of nine different scenes, three for the assessment (functionality and sense of embodiment) and six for training and familiarization. An initial room allowed the user to select among all the different scenes by holding his arm into the specific scene widget. The participants were able to move freely in each scene and were guided by the experimenter.

#### Gaming environment

**Fig. 4:**
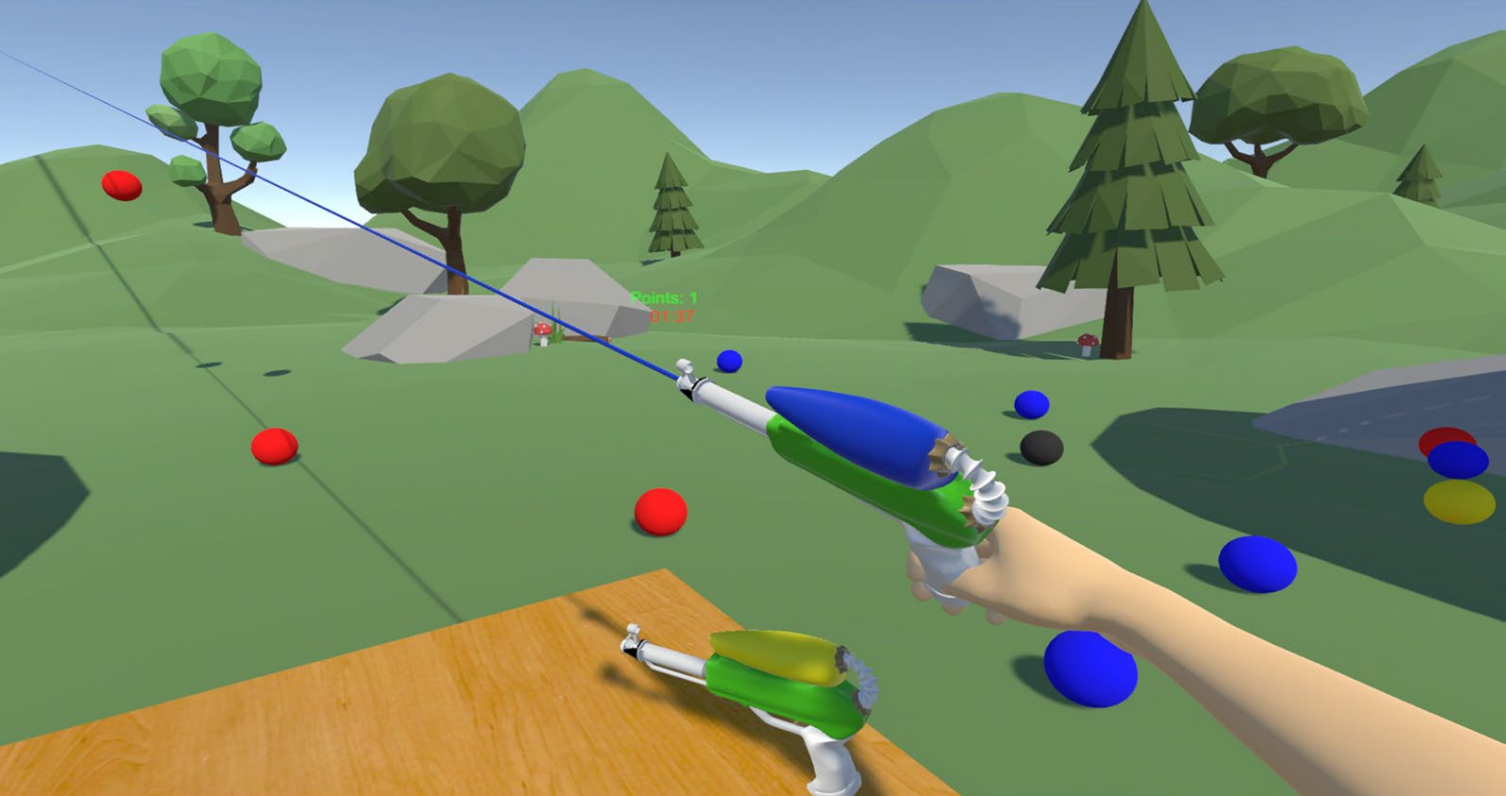
The figure presents the gaming environment which was designed and used in the familiarization phase of this study. The environment consists of a shooting game, where the user can grasp one of the three water pistols placed on the table and shoot only water balloons with a matching color. In the picture, a participant is holding a blue water pistol and pointing it toward a falling water balloon to break it

The gaming environment was designed to allow the participants to familiarize themselves with the virtual reality setup and the control method in an engaging activity. In the game, the participants were placed in a virtual natural landscape and were asked to control the virtual arm and hand to pick up one of the three water guns placed on a wooden table in front of them. Each water gun had a different color (red, yellow, and blue) and would be used to break the constantly falling water balloons with a corresponding color. If the participant hit a balloon with a color that matched the one of the water pistols, the balloon disappeared, and the user collected one point. The score and a countdown timer was shown in the top-center of the users’ field of view. Thereby the user was motivated to switch the water guns continuously in a random order to match the colors of the appearing balloons. The movements used to pick up the water pistols were forearm pro-/supination and hand opening/closing. Triggering was active as long as the water pistol was held. The user was free to move around in the scene, and balloons were spawned at ± 90^∘^ radius from the users starting position. Figure 4 shows the implemented scene.

#### Sense of embodiment assessments

**Fig. 5:**
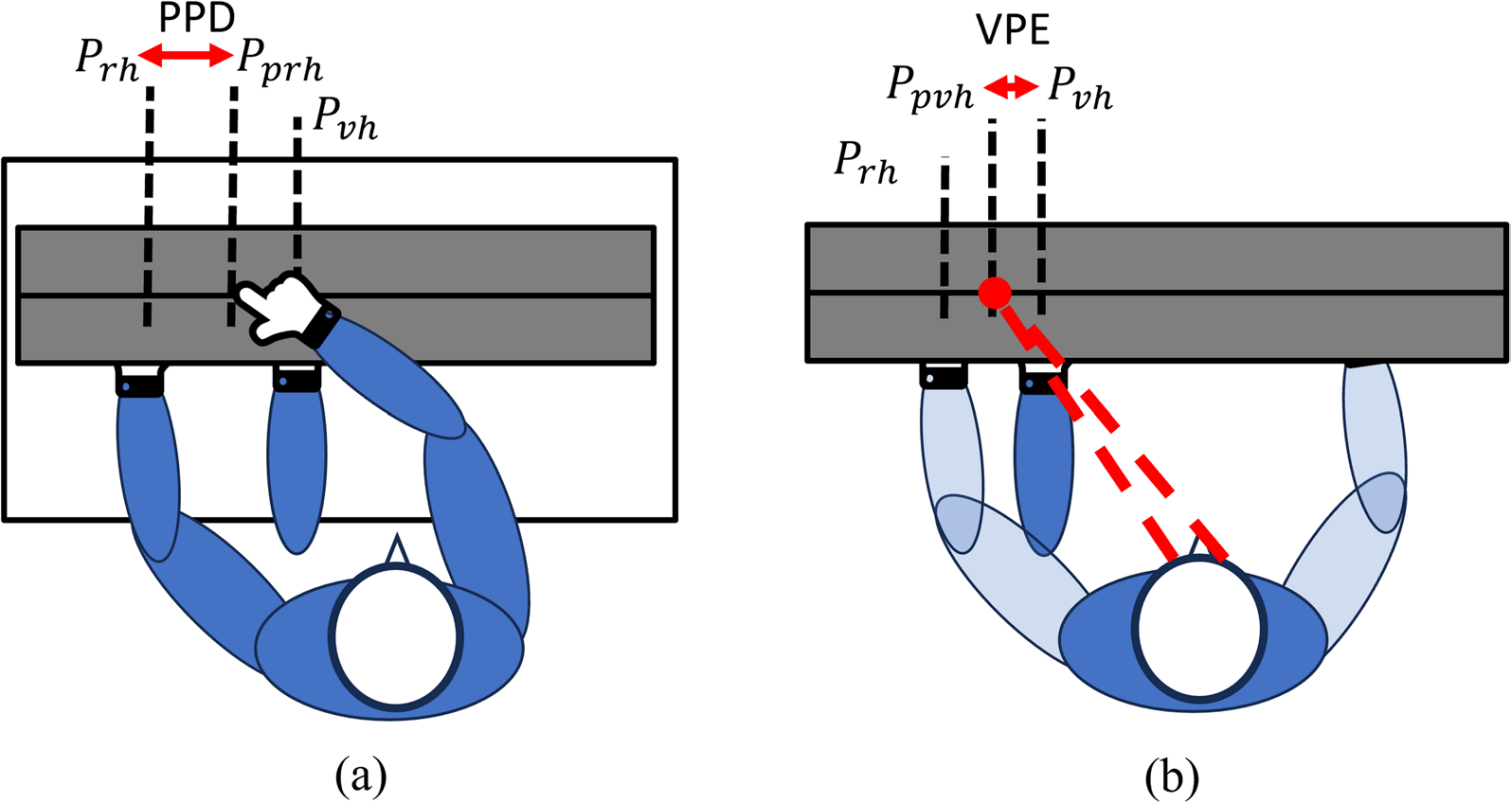
Schematic visualization of the proprioceptive drift (PPD) (**a**) and the virtual proprioception error (VPE) (**b**). The PPD is computed over the difference from the real hands position ($P_{rh}$) and where the participant thinks the real hand is ($P_{prh}$). The VPE computes the difference between the virtual hand ($P_{vh}$) and where the participant thinks the virtual hand is ($P_{pvh}$)

**(1) Virtual Arm Proprioception Error (VPE):** While there is no consensus in the community on a standardized clinical assesments to measure quantitatively the sense of embodiment [6], the proprioceptive drift (PPD, Fig. 5a) test is commonly used in literature as a quantitative measure for the sense of embodiment, e.g., especially in studies focused on the rubber hand illusion [41–43]. While this test is mostly conducted in a physical environment, previous works already implement the same principle in non-immersive VR settings by enforcing a position offset with respect to the participants real arm [27, 44].

The accurate proprioceptive awareness of our body location is fundamental for effective interaction with the environment, and a distorted representation of our arm which might hamper dexterous manipulation skills. This aspect deserves careful consideration, especially in the field of prosthetics and motor disabilities. In this work, we implemented a modified version of the PPD test for our immersive virtual environment that evaluates the self-location perception. In the original PPD test, the rubber hand ($P_{vh}$ in Fig. 5a) as well as the real hand ($P_{rh}$ in Fig. 5a) are occluded and the participants are asked to identify the perceived location of their real hand using the contralateral side or a voice command. In the PPD, the common distance between the rubber, or virtual hand lies in the range of 15–30 cm [44–46]. Unlike in the PPD, we measured the distance between the perceived position of the virtual arm (when not visible in the screen) and the actual location of the virtual arm, instead of the real arm. This was decided because our main interest lies in the proprioception evaluation of the virtual hand related to the tested condition. Hence, we did not deliberately introduce an offset distance to the position of the virtual hands in the modified version of the PPD used in our study. Nonetheless, there was still a slight discrepancy between the position of the virtual hand and the real hand of the subjects. This variability falls within a range of 5–10 cm, influenced by factors such as the individual’s lower arm length, hand dimensions, and the tolerances of the tracking system. The scene was implemented in a plain black environment to avoid potential distraction and maintain the focus on the task. The participants entered the room and saw their virtual arm for 3 s before it vanishes. From then, the only visible element was a red sphere that moved from left to right on a horizontal plane at a speed of 0.5 m/s, placed at the height of the users hand. The participants were asked to say “stop” when they perceived that the ball had reached the center position of the virtual hand (see Fig. 5b). If participants were not able to provide this information on a first trial, the test restarts immediately. The distance between the perceived virtual arm (corresponding to the location of the red ball when the participant says stop), and the actual position of the virtual hand (noted as $P_{vh}$) was then defined as the virtual arm proprioception error (VPE). We expected that the intuitive knowledge of the virtual hands position and thereby the distance between virtual hand and perceived virtual hand changes depending on the condition. The VPE was measured in Unity units, which have the scale 1 Unity unit = 1 m (100 cm). As described in section 2.4, a baseline measurement was performed immediately after entering the VR which was then included in the VPE measurements for the conditions (section 2.1) as described by Eq. 1 ($C \in$ *[baseline, LD asynchronous, LD synchronous, HD asynchronous, HD synchronous]*) and Eq. 2.

1 $$\begin{aligned} VPE_{C} = abs(P_{pvh} - P_{vh}) \end{aligned}$$

2 $$\begin{aligned} VPE = VPE_B - abs(P_{pvh} - P_{vh}) \end{aligned}$$

**(2) Questionnaire:** As an additional evaluation of the subjective embodiment, we administrated an established questionnaire, adapted from Longo et al. [34], due to its widespread familiarity and frequent use in the literature. This questionnaire is a widely used, psychometric tool based on ten statements rated on a 7-point Likert scale ranging from 1 (= strongly agree) to 7 (= strongly disagree). Similarly to the approach by Fröhner et al. [32], we adapted the items by selecting all items except item 8, which is not applicable to our study since we have no sensation of touch in the VR. The selected items are shown in Table 1. The questionnaire was implemented as one scene in the VR environment to avoid interruptions and potential reduction of the immersiveness while removing the VR HMD. This environment was designed as a plain black room with the questions displayed in the central area together with the 7-point Likert scale.

**Table 1:** Selected items (1–7, 9–10) from Longo et al. [34] to evaluate the subjective embodiment

**Ownership.** It seemed like...	
... I was looking directly at my own hand, rather than at a virtual hand.	
... the virtual hand began to resemble my real hand.	
... the virtual hand belonged to me.	
... the virtual hand was my hand.	
... the virtual hand was part of my body.	
**Location.** It seemed like...	
... my hand was in the location where the virtual hand was.	
... the virtual hand was in the location where my hand was.	
**Agency.** It seemed like...	
... I could have moved the virtual hand if I had wanted.	
... I was in control of the virtual hand.	

The *rubber hand* was replaced by the *virtual hand*

#### Training environments

**Fig. 6:**
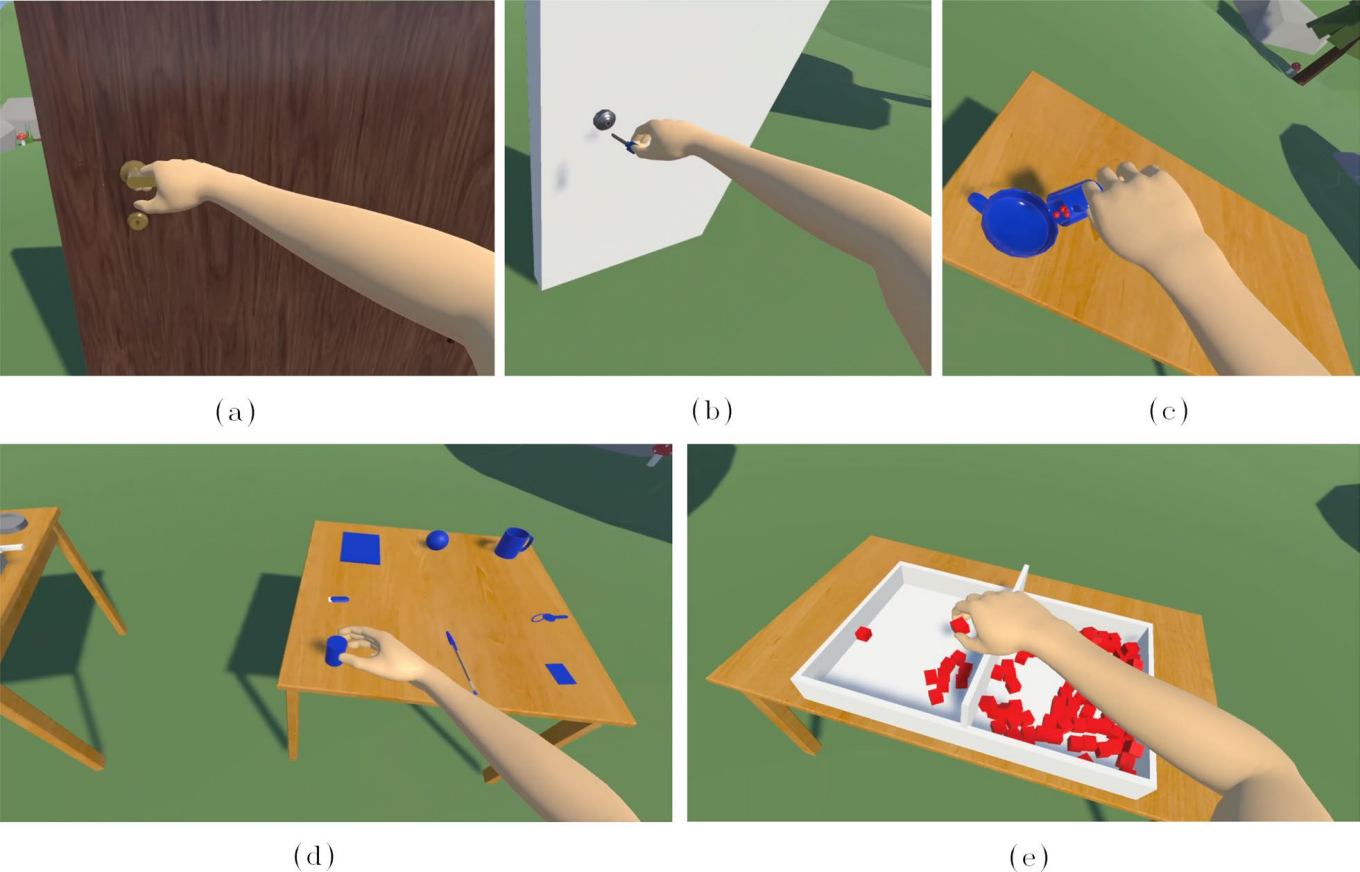
The pictures show the five different virtual training environments designed and used in this study. **a** Opening a door by grasping and turning a doorhandle. **b** Holding a key to be inserted into a lock. **c** Puring balls from a cup into a bowl. **d** “Clean Sweep” task, known from Cybathlon 2020 [40]. **e** Box and blocks environment, where users can pick and place red blocks from one box to the other

To study the effect of the training within the proposed conditions, additional VR scenes were implemented with the specific goal of encouraging the participant to explore the control method and practice. For each scene, we included tasks that require simultaneous control of hand closing/opening and forearm pro-/supination movements. The environments were inspired by activities of daily life (ADL) and standard assessment for upper-limb impairments. The five scenes implemented are presented in Fig. 6. In two of the scenes, the participant was asked to open a door. In the first case (Fig. 6a), the door had a standard door handle that should be pressed down and rotated to open the door. In the second scene (Fig. 6b), the door was locked and the key was placed on a table next to it. The participant was asked to pick up the key, insert it into the lock and rotate it by 90^∘^ to open the door. In the third scene (Fig. 6c), the participants had to grasp a cup filled with balls by the handle and pour them into a bowl placed on a table. This task focused primarily on training the full range of forearm pronation movement needed to completely empty the cup. To simulate activities of daily living in a pick and place scenario, the fourth scene reproduced the Clean Sweap task from the Cybathlon 2020 Global Edition [40] (Fig. 6d). Similarly to the physical task, this scene presented two tables and eight different objects that should be grasped from one table and placed in a specific location on a second one. Finally, the last training scene implemented a virtual version of the Box and Blocks (Holser and Fuchs 1957), where the participant was asked to move as many red blocks as possible from one box to another (Fig. 6e). In this case, we did not consider the number of blocks moved as scoring, but we let the participant play freely with the task.

#### Functionality test environment

A 3D version of the target achievement control (TAC) test [33] was implemented as a standard method to assess real-time myoelectric control functionalities. During the TAC test, participants had to move their virtual hand and forearm to randomly defined target positions. The task parameters were modified to 30 target positions for combined hand closure and wrist pro-/supination positions. The time to reach the target position was set to 15 s. If the participant was not capable to reach the position within the time frame, the target counted as failed and the next target appeared. The time the virtual hand had to be held in the target position was set to 1 s. The tolerance between the target position and the actual position was selected to 15%. The measured metrics were the success rate (SR), the time needed for the participant to reach the target position—time to target (TtT), and the accumulative time the participant was in the target position while not holding it there for 1 s—time in target (TiT).

### Statistical analysis

Data normality was evaluated by Shapiro–Wilk test. For non-parametric data, such as for the likert-scale scores in the subjective embodiment, a Quantile Transformation was applied to convert the numerical data to following a Normal distribution. The statistical analysis was performed using a repeated measure analysis of variance (ANOVA, type III), with experiment-depending parameters as factors; named as *Sensors* *Delay* and *Training*. Differences were considered statistically significant for p-values below the threshold of $\alpha$ = 0.05. A post-hoc Tukey test was performed after ANOVA to extract significance among factor interaction conditions. Means are reported as *M*: mean ± standard error.

## Results

In this section, we present the results of the embodiment metrics (VPE and subjective embodiment score), as well as the functionality measurements from the TAC metrics. The Shapiro-Wilk tests reported not normally distributed data for the questionnaire score, the VPE, the TAC test TtT, and TiT metrics (p < 0.05), and normal distributed data for the TAC SR metric (p = 0.128). After the data transformation for not normally distributed measurements, a two-way ANOVA was used to determine relevant differences for all three dependent variables, i.e., subjective embodiment score, virtual arm proprioception error and task performance.

### Embodiment results

**Fig. 7:**
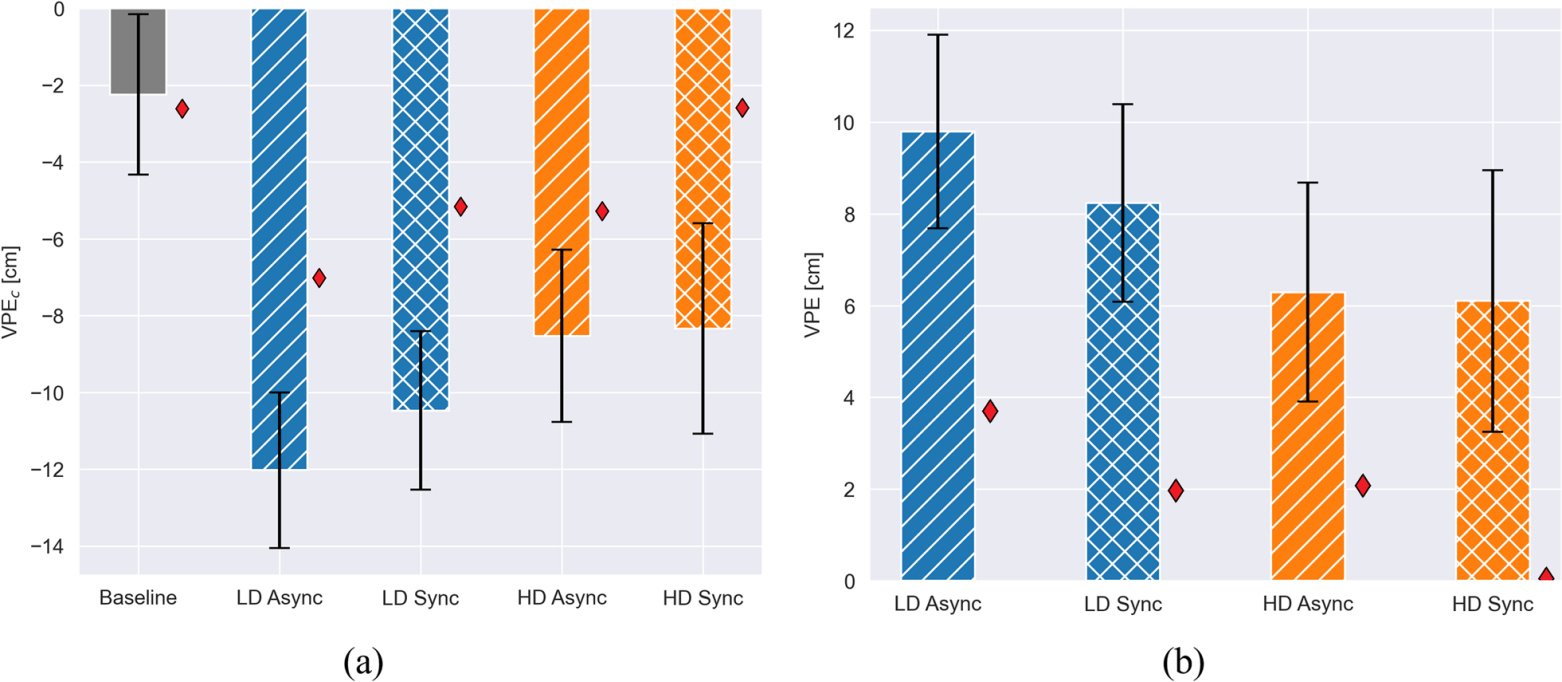
Virtual arm proprioception error (VPE). Red diamonds present outcomes from the prosthesis user, excluded from the statistical analysis and solely used for representing the target population. **a** Shows the $\hbox {VPE}_C$ for the baseline and the conditions without taking the baseline into account (Eq. 1). In **b** we visualize the VPE according to Eq. 2. Estimated means and standard errors from a post-hoc Tukey test are reported in a barplot format. The data was transformed via a quantile transformation for the statistical analysis, and its inverse transform was applied to the results presented

All statistical results for the evaluation of the Sense of Embodiment are presented in Table 2. Figure 7a presents the $VPE_C$ values without taking the baseline into account and Fig. 7b the VPE results according to Eq. 2 (the difference from each condition to the baseline). Even though without significance, a trend in smaller VPE values for the HD conditions (sync: 6.1 cm, async: 6.3 cm) compared to the LD conditions (sync: 8.2 cm, async: 9.8 cm) is observed (Fig. 7). Moreover, among LD conditions a trend of smaller VPE values is present for the synchronous condition (8.2 cm) compared to the asynchronous condition (9.8 cm). Additionally, results from the prosthesis user are displayed with red diamonds in Fig. 7. The observed differences among experimental conditions for able-bodied subjects persist for the amputee, with a more distinct contrast between *Delay* conditions, visible also in the controllers with HD. Note that $VPE_c$ is equitable in both Baseline and HD Sync.

**Table 2:** ANOVA test for embodiment metrics

Variable	Sensors	Delay	Sensors $\times$ Delay
VPE	p = 1.0, F < 0.0001	p = 1.0, F < 0.01	p = 1.0, F < 0.01
Embodiment	p $=$ 0.012, F = 6.38	p = 0.139, F = 2.19	p = 0.048, F = 3.9
Ownership	p $=$ 0.016, F = 5.86	p = 0.571, F = 0.32	p = 0.692, F = 0.16
Location	p $=$ 0.736, F = 0.11	p = 0.06, F = 3.56	p = 0.072, F = 3.28
Agency	p $=$ 0.064, F = 3.48	p = 0.679, F = 0.17	p = 0.0612, F = 3.55

Note that VPE refers to Virtual Arm Proproception Error, and Embodiment, Ownership, Location and Agency are the subjective embodiment scores from the questionnaire (Longo et al. [34])

**Fig. 8:**
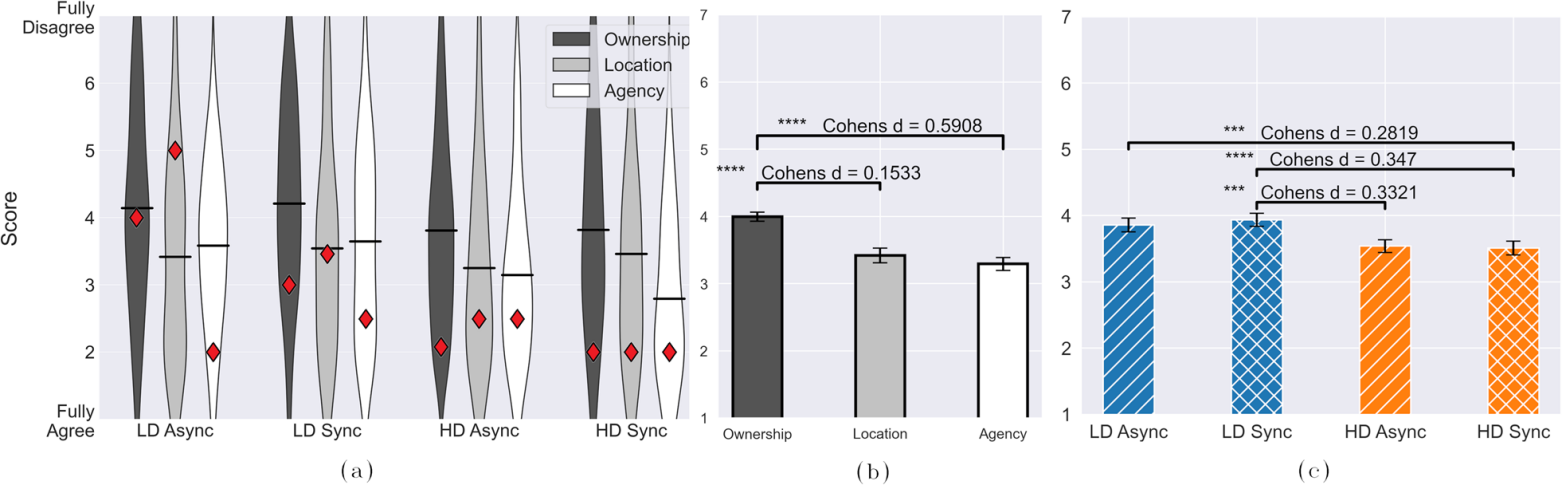
Subjective embodiment results from the questionnaire. **a** Displays the distribution of the experimental data, separated into the three components of the survey: ownership, location and agency. Data is presented in violin plots with their respective mean. Red diamonds reports results from the prosthesis user, excluded from the statistical analysis and solely used for representing the target population. **b** displays the estimated means and standard errors from a post-hoc Tukey test, with asterisks that mark significance between components, together with the Cohens distance *d*. **c** reports the estimated means and standard errors from a post-hoc Tukey test, with asterisks that mark significance between tested conditions. The lower the score, the stronger the participants agreed with the items from Table 1, which indicates a stronger embodiment

Regarding subjective experience, Table 2 reports *Embodiment*, which represents the questionnaire ratings for all 9 Items for each participant and condition. To examine where exactly the differences between conditions are, we analyze scores provided to the subscales *Ownership*, *Location*, and *Agency*. Four ANOVA Test with the factors *Sensors* and *Delay* were calculated to explore whether the conditions differ from each other. Contrary to the results of the VPE, the number of sensors resulted significant (p $=$ 0.012) for the subjective embodiment score. Furthermore, while no significance is obtained regarding the factor *Delay*, the interaction between *Sensors* and *Delay* (p $=$ 0.048) shows a significant effect, with results reported in Fig. 8c. Significantly higher subjective embodiment, i.e., lower score, is shown in condition **C4** (HD sync; *M*: 3.509 ± 0.105), compared to conditions **C1** (LD async; *M*: 3.856 ± 0.102) and **C2** (LD sync; *M*: 3.935 ± 0.1), as well as in condition **C3** (HD async; *M*: 3.537 ± 0.096) with respect to **C2** (LD sync). Overall, the VPE presents a lower estimated mean for the HD condition (6.1 cm, 6.3 cm) than for the LD condition (8.2 cm, 9.8 cm), which is in alignment with the stronger subjective embodiment for HD (*M* = 3.523 ± 0.071) than for LD (*M* = 3.896 ± 0.071), as can be seen in Figs. 7 and 8c, respectively.

Figure 8a presents the answers to the subjective embodiment survey separated among the three subscales: Ownership, Location and Agency, and according to each condition tested **C1–C4**. Table 2 proves that the number of sensors are significantly relevant for the ownership components. Results of the corresponding post-hoc Tukey test are presented in Fig. 8b to compare scores from the three subscales. These show significantly higher score (p < 0.0001) between perceived ownership (*M*: 4.08 ± 0.069) and location (*M*: 3.255 ± 0.09), as well as between perceived ownership and agency (*M*: 3.24 ± 0.102). Figure 8a reports also results from the prosthesis user with red diamonds. Differences among experimental conditions are more pronounced compared to results from able-bodied subjects, with LD Async obtaining the worst scores and HD Sync the best. Although HD exhibits consistent scores among subcomponents of embodiment, variations are observed in LD, underscoring how sEMG interfaces have a strong relationship with Agency.

### Functional results

For the third dependent variable, regarding task performance, three two-way ANOVA were calculated. Table 3 reports their results according to the three TAC metrics accounting for the assessment of functionality: success rate (SR), time to target (TtT) and time in target (TiT). Better task performance is associated to higher SR, lower TtT and higher TiT. In this case, factors included in the analysis are: number of sensors (*Sensors*), pre- and post-training outcome (*Training*), and their interaction (*Sensors* $\times$ *Training*). Note that for the functionality assessment the number of participants experiencing each control condition is N = 12.

**Table 3:** ANOVA test for functional metrics: success rate (SR), time to target (TtT) and time in target (TiT)

Variable	Sensors	Training	Sensors $\times$ Training
TAC SR	p $=$ 0.012, F = 13.36	p = 0.307, F = 1.07	p = 0.915, F = 0.01
TAC TtT	p $=$ 0.061, F = 3.71	p $=$ 0.584, F = 0.303	p = 0.28, F = 1.18
TAC TiT	p = 0.259, F = 1.31	p = 0.514, F = 0.43	p = 0.538, F = 0.39

The factor Training is representative for pre- and post-training

**Fig. 9:**
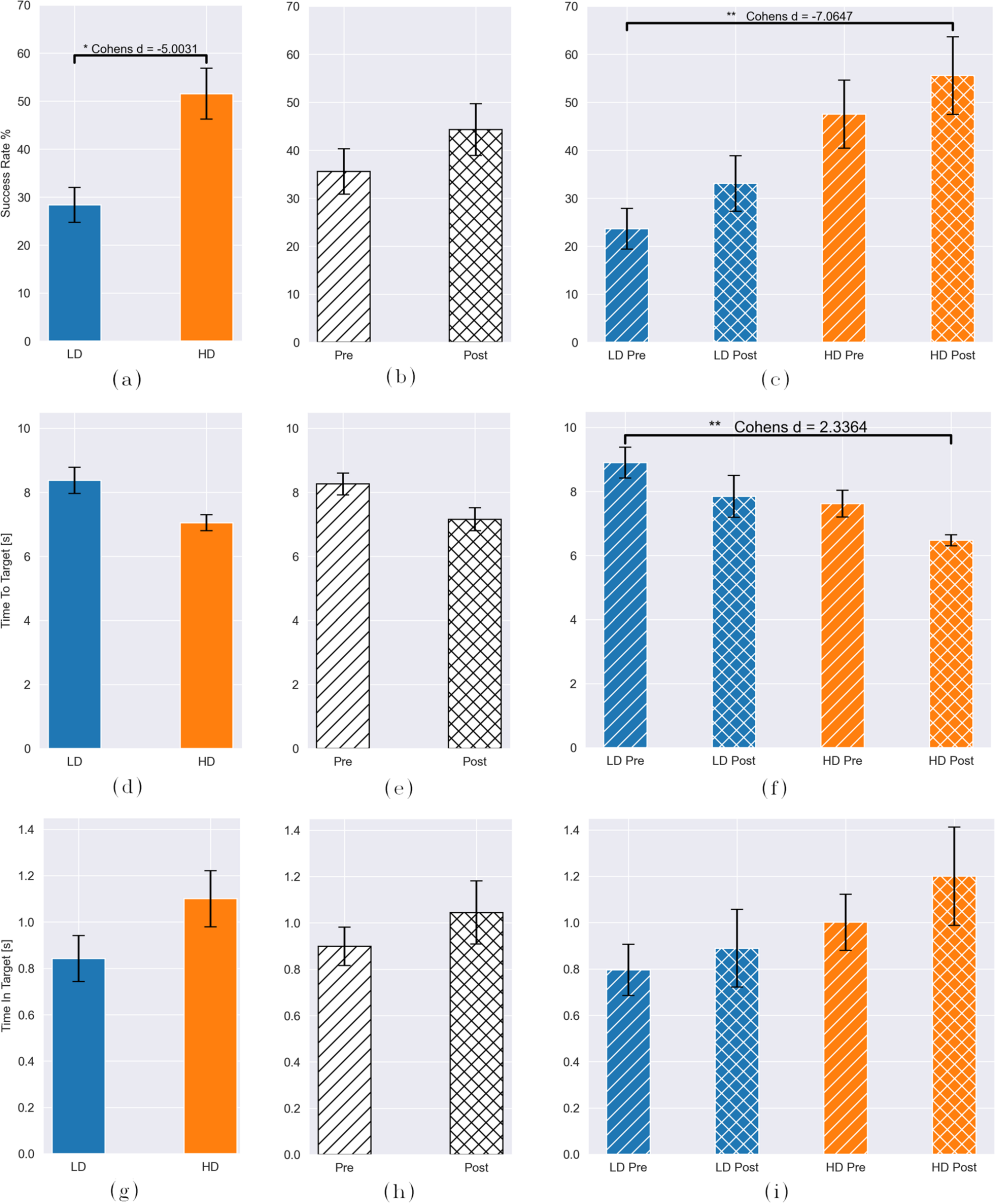
Functional results from the target achievement control (TAC) test. TAC metrics are reported in row-wise groups: Success rate in **a**–**c**, time to target in **d**–**f**, and accumulative time in target in **g**–**i**. For all metrics, column-wise groups present results for different factors and their interactions: panels (**a, d, g**) shows the results for *Sensors*: conditions (C2) vs (C4); panels (**b, e, h**) for *Training*; and panels (c,f,i) for *Sensors x Training*. All panels display the estimated means and standard errors from a post-hoc Tukey test, with asterisks that mark significance between conditions, together with the Cohens distance *d*. LD = low density; HD = high density; pre = pre-training; post = post-training

Significance is present on the Success Rate (SR) and in relation to the number of sensors (p $=$ 0.012, see Fig. 9a), with LD reporting a $M_{SR}$ of 28.33% ± 3.639 and HD a $M_{SR}$ of 51.53% ± 5.324. Further statistical significance (p < 0.01, see Fig. 9c) is observed between **C2** (LD) pre-training ($M_{SR}$: 23.61% ± 4.233) and **C4** (HD) post-training ($M_{SR}$: 55.6% ± 8.096). A non-significant trend in higher SR scores is visible from Fig. 9b for the training factor.

Regarding the time to reach the target (TtT), the ANOVA results showed no significance for both *Sensors* (p $=$ 0.061) and *Training* (p $=$ 0.584) factors. Nonetheless, HD and post-training conditions showed better timing performance, with $M_{TtT}$: 8.371 s ± 0.413 for LD, $M_{TtT}$: 7.046 s ± 0.251 for HD and $M_{TtT}$: 8.26 s ± 0.34 for pre-training, $M_{TtT}$: 7.157 s ± 0.36 for post-training (see Fig. 9d and e). The interaction between factors (Fig. 9f) showed higher TtT for **C2** (LD) pre-training ($M_{TtT}$: 8.899 s ± 0.483) compared to **C4** (HD) post-training ($M_{TtT}$: 6.471 s ± 0.169) (p < 0.01).

Results from the time in target (TiT) are presented in Fig. 9g–i. Even though no significant differences are obtained among factors or conditions, we observed a similar performance trend to TtT, with longer times (better performance) in the consecutive order from **C2** (LD) pre-training to **C4** (HD) post-training, as well as from **C2** (LD) to **C4** (HD) and pre- to post-training individually.

**Fig. 10:**
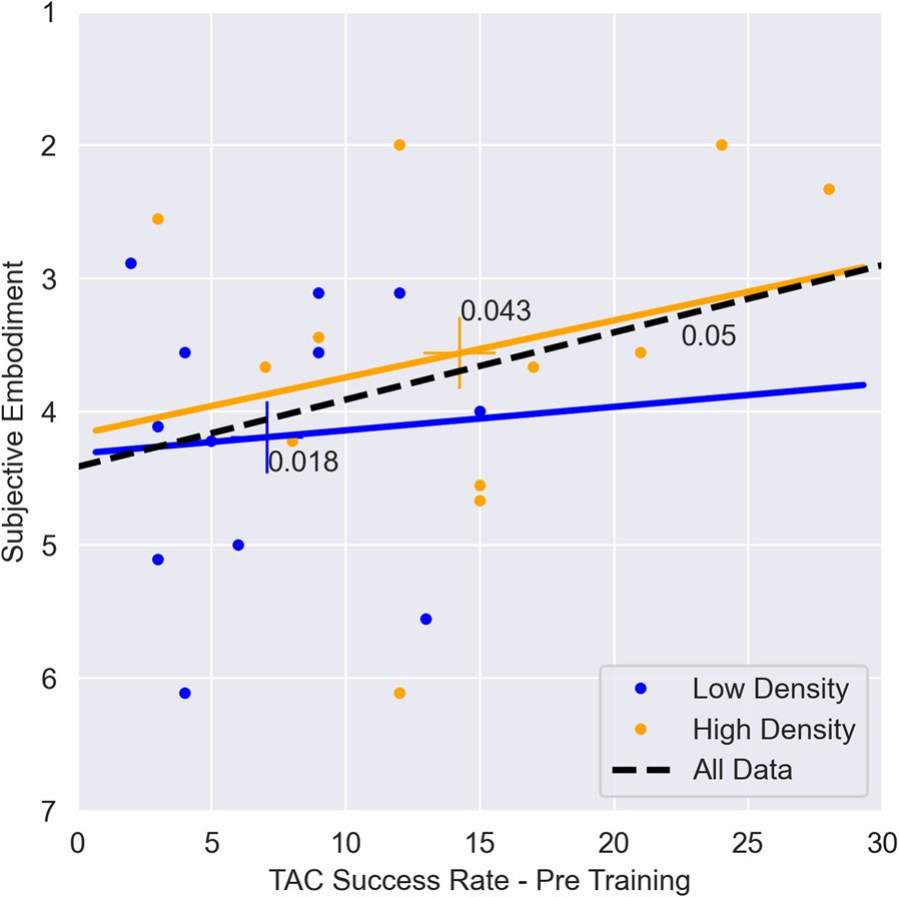
Correlation between subjective embodiment and functionality metric. TAC Success Rate is selected as most representative functional metric for this study. Note that the TAC values correspond to the Pre training session, conducted immediately after participants provided their responses regarding the subjective embodiment following the Interaction Phase with the tested controller (see Fig. 1). Values proximal to the regression line indicate the slope of their relationship

Finally, we investigate the relation between the changes in functionality through high and low density interfaces and subjective embodiment. Figure 10 shows the result of plotting the subjective embodiment against the TAC SR before training (representative for the functionality closest to during conditions), for LD (blue) and HD (orange). Participants using the HD interface achieved higher values of both aspects, with a higher slope (0.043) compared to the LD interface (0.018). This indicates a positive relationship between the higher subjective embodiment percevied through a higher functionality.

## Discussion

Only non-significant variations in the virtual arm proprioception error (VPE) were observed across the different tested conditions (Fig. 7 and Table 2). This might be due to the small distance between the virtual hand and the real hand position which was intentionally not set to distinct values as occures in the classic PPD, and thereby limits the phyiscal possible drift of the virtual hand dependent to the condition. Furthermore, the absence of contextual cues in immersive VR environments may negatively impact participants proprioception, as raised in [47], and decrease the measurable effect in the VPE.

Regarding the subjective embodiment, the number of sensors showed a significant effect. Participants experienced a stronger subjective embodiment for high density with respect to low density control methods, independently of the delay implemented. Contrary to what we hypothesized in **H3**, and what other studies have found [32, 48], the time delays did not present a strong effect. For instance, LD synchronous reports the highest score (lowest embodiment) in Fig. 8c, suggesting a potential inability of perceiving a time delay when the control quality is poorer. Furthermore, the differences between the synchronous and asynchronous control conditions might be explained by the used control modality. In our study, the position of the prostheses is simultaneously and proportionally controlled with sEMG interphases, usually less precise than other control modalities, such as infrared cameras [27] and tracking or mechanical manipulators [32]. Even though sEMG are intuitive solutions for human–machine interfacing, when controlling a system through biosignals, there is no specific feedback from the control source to the user. Thereby the user may not be aware if the delay occurs because of the control algorithm or because of the condition. The results from the TAC metric TtT (in Fig. 9d–f supports this explanation, as we can observe that the average time the participants require to reach a desired position is 7 s–8.2s (LD, HD, respectively, and consistent with [35] for 2 DoFs). Even though the implemented delay of 500ms should be recognizable [36], the TtT results are considerably higher than this.

Results depicted in Fig. 8b demonstrated higher agreement, i.e. lower score, with the location and agency components of the survey compared to the ownership component. Our findings of a stronger perceived Agency component (see Fig. 8b), and the Ownership being the weakest perceived component are in alignment with the observations from [30]. Considering the Agency component (Fig. 8a), we observe that only for HD conditions, the synchronous condition achieve a lower score, corresponding to a stronger subjective embodiment. Overall, it is intuitive to perceive a change in Agency from a time delay since it directly affects the responsiveness of the control.

These results highlight the persistent challenges associated with perceiving an external arm as one’s own in VR environments, independently of the sEMG configuration used. Finally, for this preliminary study, the physiological dimensions of the virtual arm, as well as its positioning in reference to the HMD, are not adapted to the individual participant which might have an influence on the Ownership and the Location components.

While the results from the prosthesis user are anecdotal in nature, they serve to underscore and emphasize the statistical findings observed in able-bodied subjects. Note that these results often highlight apparent differences among experimental conditions, both concerning the number of sensors and the implemented delay in the myoelectric controller. Despite preliminary, these outcomes indicate the potential impact that sEMG interfaces may have on the embodiment of a virtual prosthesis.

These results underscore the intimate connection of this experiment design with the subcomponent agency, illustrating how the chosen embodiment test, along with testing conditions and/or protocol, strongly influences a portion of the overall embodiment experience for the prosthesis user.

Results from TAC demonstrated functional enhancements associated with an increased number of sEMG sensors employed. In fact, as we hypothesized, we obtain significantly higher SR and lower TtT values for condition HD compared to LD. These results align with existing literature on myoelectric control [49]. Moreover, when combined with our significant results on subjective embodiment, which also relates to the number of sensors, it is reasonable to assume that augmented functionality contributes to the sense of embodiment in a VR environment. Although anecdotal, Fig. 10 also demonstrates an increasing subjective embodiment with higher functionalities irrespective of the sensor configuration (LD or HD), and with larger effect for HD sensors which supports our hypothesis **H4**. This assertion is further supported by [6], which emphasizes the strong association between agency, and volition. Furthermore, we observed an immediate impact of VR training, as evidenced by improved time-to-target performance (Fig. 9b, e, and h). Note that participants already experienced VR for 10 min during the embodiment phase of the experimental protocol, followed by a 10-minute training during the functional phase.

## Conclusion

In this study, we investigated the impact of different sEMG configurations and delay on the sense of embodiment and functional outcomes in an immersive virtual reality setup. Results from the functional assessment demonstrated that higher success rates are achieved by employing a higher number of sEMG sensors (named HD condition). Likewise, results from the questionnaire shows enhanced subjective embodiment during the HD condition. Albeit no significant differences among conditions, the virtual arm proprioceptive error (VPE) for LD sEMG sensors achieved smaller values in the synchronous condition compared to the asynchronous condition. Even if the performance of our myoelectric control is in alignment with the state of the art, the absence of significant changes in response to delayed control highlights the persisting limitations in developing simultaneous and continuous myoelectric control solutions. While this study has been conducted using a regression algorithm, future studies will investigate the effect of different myoelectric control methods and their impact in the Sense of Embodiment. Regarding the use of virtual reality environments as a testing platform, all functional metrics showed an immediate functional improvement (without significance) after training in VR environments. Nevertheless, the non-individualized anthropometry dimensions of our VR environment may reduce the general embodied experience and in particular, any location-based metric. Therefore, the adaptation of the proprioceptive drift experiment into immersive VR environments should be further explored. In summary, the combination of functional metrics with subjective embodiment scores provides a preliminary outcome suggesting the positive relationship between both aspects. Limiting our experimentation to able-bodied subjects overcomes issues with the varied and pathological muscle conditions exhibited by the population of prosthesis users. While this approach also facilitates the inclusion of a greater number of participants for statistical analysis, it comes at the expense of restricting the applicability of our conclusions to the target population. Nevertheless, we conducted the same experimental protocol with a prosthesis user, aiming to investigate the potential generalization of our results to real end-users. Recent promising results of machine learning myoelectric control algorithms, along with their incorporation into commercial systems, emphasize the importance of extending this evaluation to a larger group of participants with limb loss. The study shows the significance of sEMG interfaces into both function and sense of embodiment in immersive virtual reality. Results on able-bodied subjects reveal a positive relationship between performance and subjective embodiment, calling for further investigations to advance the natural integration of prostheses.

## Data Availability

All relevant data is within the paper. Detailed information may be available from the corresponding author after request.
