# Correction: Experimental evaluation of the impact of sEMG interfaces in enhancing embodiment of virtual myoelectric prostheses

**DOI:** 10.1186/s12984-024-01368-z

**Published:** 2024-04-27

**Authors:** Theophil Spiegeler Castañeda, Mathilde Connan, Patricia Capsi-Morales, Philipp Beckerle, Claudio Castellini, Cristina Piazza

**Affiliations:** 1 Department of Computer Engineering, Technical University of Munich (TUM), Garching bei Munich, Germany; 2 Institute of Robotics and Mechatronics, German Aerospace Center (DLR), Oberpfaffenhofen, Germany; 3 Munich Institute of Robotics and Machine Intelligence, Technical University of Munich (TUM), Munich, Germany; 4 Department Artificial Intelligence in Biomedical Engineering, Friedrich-Alexander-Universität Erlangen- Nürnberg (FAU), Erlangen, Germany; 5 Department of Electrical Engineering, Friedrich-Alexander-Universität Erlangen-Nürnberg (FAU), Erlangen, Germany

Journal of NeuroEngineering and Rehabilitation (2024) 21:57

10.1186/s12984-024-01352-7

Following publication of the article [1], figure citation was incorrectly given as (see Fig. 1) but should have been (see Fig. 2) under the sub-heading EMG setup and data collection.

The original article has been corrected.
